# Two New Monoterpenes from the Fruits of *Illicium lanceolatum*

**DOI:** 10.3390/molecules181011866

**Published:** 2013-09-26

**Authors:** Ji-Feng Liu, Hui-Juan Li, Li-Xia Wang, Meng-Qi Liu, Yue-Feng Bi, Yan-Bing Zhang

**Affiliations:** 1 School of Pharmaceutical Science, Zhengzhou University, Ke Xue Da Dao 100, Zhengzhou 450001, China; 2 School of Pharmaceutical Science, Henan University of Traditional Chinese Medicine, Zhengzhou 450008, China

**Keywords:** *Illicium lanceolatum*, monoterpenes, anti-microbial activity

## Abstract

Two new monoterpenes, *p*-mentha-1(7),8-dien-2-*O*-*β*-d-glucoside (**1**) and *trans*-2,4-dihydroxy-2,4-dimethyl-*trans*-1-acetic acid *γ*-lactone (**2**) were isolated from the fruits of *Illicium lanceolatum* along with *trans*-2,4-dihydroxy-2,4-dimethyl-*cis*-1-acetic acid *γ*-lactone (**3**), (1*R*,2*R*,4*R*)-8-*p*-menthen-1,2-diol (**4**), *trans*-sobrerol (**5**), (1*S*,2*S*,4*R*)-*p*-menthane-1,2,8-triol (**6**) and (1*S*, 2*S*, 4*R*, 8*R*)-*p*-menthane-1,2,9-triol (**7**)*.* The structures of the isolates were confirmed by spectroscopic analysis and they showed no inhibitory effects on the *in vitro* growth of microbial organisms (*Escherichia coli*, *Staphyloccocus aureus*, *Bacillus subtilis*) at less than 1.0 mg/mL.

## 1. Introduction

**Figure 1:**
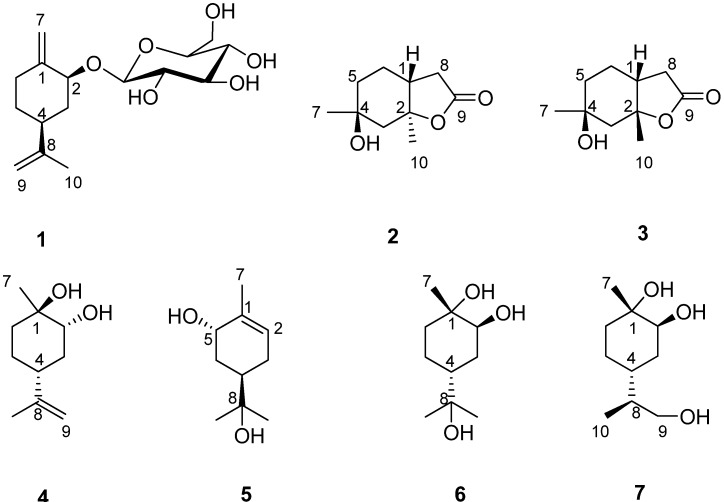
Structures of compounds **1**–**7**.

The *Illicium* (Illiciaceae) genus consists of aromatic evergreen trees that are distributed primarily in southwestern China and the southeast of America [1]. Investigations on the chemical constituents of *Illicium* have led to the isolation of monoterpenoids, sesquiterpenoids, phenylpropanoids, lignans and flavonoids, some of which exhibited anti-bacteria, neurotoxic and neurotrophic activities [2]. *Illicium lanceolatum* is a medicinal plant of the genus *Illicium* with the Chinese name ‘Mangcao’ or ‘Hongduhui’. Its roots and leaves have anti-inflammatory and analgesic activities and have been used to treat bruises, internal injuries and back pain [3]. Previous investigations of *I. lanceolatum* have resulted in the isolation of sesquiterpenes, phenylpropanoids, lignans and flavones [4,5]. As part of investigations on the genus *Illicium* to seek more novel bioactive compounds, we carried out an extensive chemical study on *I. lanceolatum*, which led to the isolation of seven monoterpene compounds (Figure 1). Among them**, 2** and **3** are a pair of stereoisomers, the latter being a known compound synthesized by Wolinsky in 1966 [6], however, the complete NMR data has not been reported to date. In this paper, we report the isolation and structure elucidation of two new monoterpenes, *p*-mentha-1(7), 8-dien-2-*O*-*β*-d-glucoside (**1**) and *trans*-2,4-dihydroxy-2,4-dimethyl-*trans*-1-acetic acid *γ*-lactone (**2**) from the fruits of *I. lanceolatum*. The spectroscopic data of *trans*-2,4-dihydroxy-2,4-dimethyl-*cis*-1-acetic acid *γ*-lactone (**3**) is also reported.

## 2. Results and Discussion

**Figure 2:**
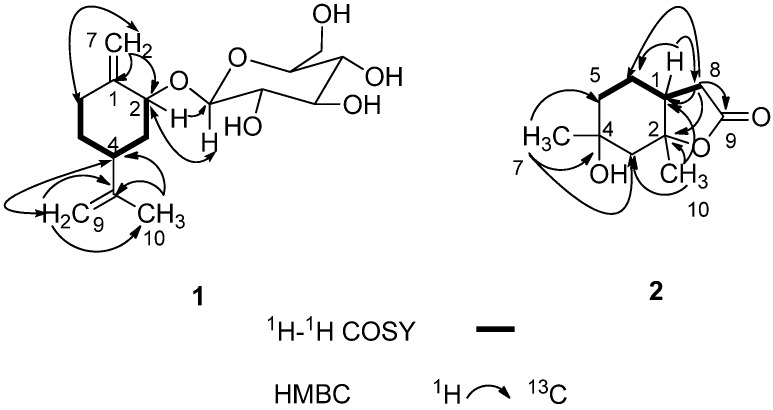
Key ^1^H-^1^H COSY and HMBC correlations of compounds **1**–**2**.

**Figure 3:**
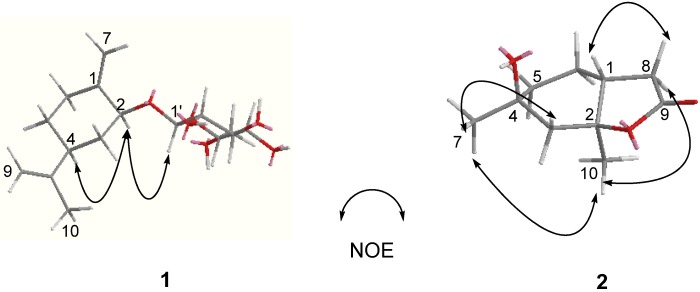
Key NOE correlations in the NOESY spectrum of compounds **1**–**2**.

Compound **1** was obtained as a white amorphous powder with the molecular formula C_16_H_26_O_6_ according to HRESIMS. The pseudo-molecular ion at 337.1622 [M + Na]^+^ (calcd. for C_16_H_26_O_6_Na, 337.1627), suggests the presence of four degrees of unsaturation. The IR spectrum showed a strong absorption band due to hydroxyl (3,423 cm^−1^) groups. The ^1^H-NMR spectrum of **1** showed the presence of one methyl group at *δ*_H_ 1.71 (3H, s); one anomeric proton signal of *β*-glucopyranosyl moiety at *δ*_H_ 4.40 (1H, d, *J* = 7.8 Hz), indicating the existence of a *β*-d-linkage sugar moiety in compound **1**; one exocyclic methylene protons at *δ*_H_ 4.77 and 5.27 (each s) (Table 1). The ^13^C-NMR spectrum of **1** displayed 16 carbon signals grouped by DEPT experiment into one methyl, six methylene, seven methine and two quaternary carbons, which consisted of a set of *β*-d-glucopyranosyl unit and a monoterpenoid unit signals (Table 1). Analysis of the ^1^H–^1^H COSY (Figure 2) and HMBC spectra of **1** led to the fragment -CH(2)-CH_2_(3)-CH(4)-CH_2_(5)-CH_2_(6) in its structure. The planar structural skeleton of **1** was further established on the basis of HMBC spectral data (Figure 2) in which the correlations between H-7 with C-1, C-2, C-6, H-9 with C-4, C-8, C-10, and H-10 with C-4, C-8 were displayed. The *β*-d-glucopyranosyl unit linked at C-2 was further supported by the correlations H-1'/C-2 and H-2/C-1' in the HMBC spectrum (Figure 2). The relative configuration of **1** was determined from its NOESY spectrum (Figure 3) in which the correlation between H-2 and H-4 was found, indicating that H-2 and H-4 were in *α* orientation. Therefore, the structure of **1** was elucidated as Figure 1 and named as *p*-mentha-1(7), 8-dien-2-*O*-*β*-d-glucoside (**1**).

**Table 1:** ^1^H- (400 MHz) and ^13^C-NMR (100 MHz) data (CD_3_OD) of compound **1**. Chemical shifts *δ* in ppm relative to TMS, *J* in Hz.

Position	δ_C_	δ_H_	Position	δ_C_	δ_H_
1	147.9	-	8	148.7	-
2	76.7	4.29 (m)	9	108.1	4.70 (s)
3	40.5	2.16–2.19 (m)			4.69 (d, 1.8)
		1.29–1.39 (m)	10	19.6	1.71 (s)
4	44.1	2.24–2.28 (m)	1'	101.8	4.40 (d, 7.8)
5	33.7	2.39–2.44 (m)	2'	74.1	3.23–3.28 (m)
		2.01–2.09 (m)	3'	77.5	3.27–3.36 (m)
6	32.9	1.78–1.81 (m)	4'	70.3	3.26–3.34 (m)
		1.13–1.18 (m)	5'	76.5	3.22–3.27 (m)
7	104.9	5.27 (s)	6'	61.4	3.87 (dd, 12.0, 2.0)
		4.77 (s)			3.65 (dd, 11.9, 5.6)

Compound **2** was isolated as a white powder. The HRESIMS of **2** showed a pseudo-molecular ion at *m/z* 207.0999 [M + Na]^+^ (calcd. for C_10_H_16_O_3_Na, 207.0997), consistent with the molecular formula C_10_H_16_O_3_. The IR spectrum suggested the presence of hydroxyl (3,447 cm^−1^) and carbonyl (1,746 cm^−1^) groups. The ^13^C-NMR and DEPT spectra displayed 10 carbon signals, including two methyl, four methylene, one methine and three quaternary carbon signals (Table 2). ^1^H- and ^13^C-NMR spectra showed two methyl groups at *δ*_H_ 1.32 (3H, s)/*δ*c 33.4, *δ*_H_ 1.48 (3H, s)/*δ*c 19.5 and the characteristic signals of a *γ*-lactone moiety at *δ*_H_ 2.37–2.42 (dd, *J* = 8.4, 16.5 Hz, H-8a), 2.43–2.49 (dd, *J* = 8.4, 16.4 Hz, H-8b) and *δ*_C_ 86.2 (s, C-2), 33.3 (t, C-8), 176.6 (s, C-9) (Table 2). The ^1^H–^1^H COSY spectrum showed cross-peaks at H-5/H-6, H-6/H-1 and H-1/H-8, indicating the presence of a structural fragment CH_2_(5)-CH_2_(6)-CH(1)-CH_2_(8) as shown with bold line in Figure 2. The planar structural skeleton of **2** was further established on the basis of its HMBC spectrum (Figure 2), in which ^1^H–^13^C long-range correlation signals were observed at H-7/C-3, C-4, C-5; H-10/C-1, C-2, C-3; H-8/C-1, C-2, C-6, C-9 and H-1/C-6, C-8. This NMR data were very similar to that of *trans*-2,4-dihydroxy-2,4-dimethyl-*cis*-1-acetic acid *γ*-lactone (**3**) synthesized by Wolinsky in 1966 [6], and the molecular formulas of the two compounds were the same, which indicated that compounds **2** and **3** were likely stereoisomers of each other. The correlation between CH_3_-7 and CH_3_-10 observed in the NOESY spectrum (Figure 3), in addition to the absence of correlation between H-1 with CH_3_-10 disclosed that H-1 is in the *β* configuration, whereas CH_3_-7 and CH_3_-10 are in the *α* configuration. Therefore, H-1 and CH_3_-10 of compound **2** was determined to have a *trans* relationship and named as *trans*-2,4-dihydroxy-2,4-dimethyl-*trans*-1-acetic acid *γ*-lactone. From a biogenetic point of view, compounds **2** and **3** perhaps came from the same precursor, (1*R*, 2*R*, 4*R*)-8-*p*-menthen-1,2-diol (**4**), according to the reference [6]. In this literature, Wolinsky and Chan reported that compound **3** came from 3-isopropenyl-6-oxoheptanoic acid which is the oxidation product of compound **4**.

**Table 2:** ^1^H- (400 MHz) and ^13^C-NMR (100 MHz) data (CDCl_3_) of compounds **2** and **3**. Chemical shifts *δ* in ppm relative to TMS, *J* in Hz.

Position	2		3	
	δ_C_	δ_H_	δ_C_	δ_H_
1	47.2	2.01–2.07 (dddd, 3.3, 7.8, 11.8, 15.2)	39.8	2.36–2.41 (m)
2	86.2	-	86.1	-
3	48.9	2.12 (d, 13.1.)	46.3	1.89 (d, 14.5)
		1.80 (d, 13.1)		1.73 (d, 14.5)
4	71.3	-	69.5	-
5	40.4	1.81–1.86 (m)	33.2	1.56–1.60 (m)
		1.53–1.60 (m)		1.48–1.55 (m)
6	21.3	1.70–1.74 (m)	21.7	2.01–2.07 (m)
		1.60–1.67 (m)		1.49–1.53 (m)
7	33.4	1.32 (s)	31.1	1.30 (s)
8	33.3	2.43–2.49 (dd, 8.4, 16.4)	34.0	2.53–2.60 (dd, 7.9, 16.8)
		2.37–2.42 (dd, 8.4, 16.5)		2.40–2.48 (dd, 7.9, 16.8)
9	176.6	-	176.3	-
10	19.5	1.48 (s)	27.4	1.57 (s)

Compounds **3**–**7** were identified as *trans*-2,4-dihydroxy-2,4-dimethyl-*cis*-1-acetic acid *γ*-lactone (**3**) [6], (1*R*, 2*R*, 4*R*)-8-*p*-menthen-1,2-diol [7], *trans*-sobrerol (**5**) [8], (1*S*, 2*S*, 4*R*)-*p*-menthane-1,2,8-triol (**6**) [9], (1*S*, 2*S*, 4*R*, 8*R*)-*p*-menthane-1,2,9-triol (**7**) [10], respectively, by comparison of their data (MS and NMR) with those in the literature.

The isolates were preliminarily evaluated against the test organisms (*Escherichia coli*, *Staphyloccocus aureus* and *Bacillus subtilis*) *in vitro*. A broth microdilution method was used to determine the minimum inhibitory concentration (MIC) [11], however, no compound was bacteriocidal against all three bacteria at less than 1.0 mg/mL.

## 3. Experimental

### 3.1. General

Optical rotations were determined on a Perkin-Elmer model 341 and Polar 3001. IR (KBr) spectra were recorded on a PE-1710 FT-IR spectrometer. 1D and 2D NMR spectra were recorded on Bruker DPX-400 NMR with TMS as internal standard. HR-ESI-MS spectra were run on a Waters-Q-Tof MS instrument. Silica gel (200–300 mesh) for column chromatography was obtained from Qingdao Meigao Chemical Company (Qingdao, China). MCI gel (10 μm) was purchased from Merck Chemicals Ltd. (Nottingham, UK). Sephadex LH-20 (20–150 μm) was purchased from Pharmacia Fine Chemical Co. Ltd., Uppsala, Sweden.

### 3.2. Plant Material

The fruits of *Illicium lanceolatum* were collected in the Tianmu Mountains, Jiangsu Province, China, in September 2011, and identified by Associate Professor Dr. Mengqi Liu from the School of Pharmaceutical Science, Henan University of Traditional Chinese Medicine. A voucher specimen (2011-09-01) was deposited in the School of Pharmaceutical Science, Zhengzhou University.

### 3.3. Extraction and Isolation

The powdered fruits of *I. lanceolatum* (5.0 kg) were extracted with 95% EtOH (20 L) under reflux for three times, 2 h each time. The extract was concentrated under reduced pressure to give a residue. The residue was dissolved in H_2_O and then extracted successively with CHCl_3_ (each 2 L) and *n*-BuOH (each 2 L). The CHCl_3_ fraction (90 g) was subjected to silica gel chromatography (1,000 g, 200–300 mesh) and eluted with a CHCl_3_/MeOH (100:0, 90:10, 80:20, *v/v*, each 8 L) gradient to afford eight fractions (Frs.1–8). Fr.3 (15 g) was further separated to obtain six sub-fractions (Frs.3a–f). Fr.3b (1.0 g) was subjected to silica gel column (40 g, 200–300 mesh) with an eluent of petroleum ether/Me_2_CO (80:20) to yield compounds **3** (10 mg) and **4** (30 mg). Fr.3c (2.0 g) was chromatographed through a silica gel column (80 g, 200–300 mesh) eluted with petroleum ether/EtOAc (90:10, 80:20, 70:30, *v/v*, each 1.0 L) to yield compounds **2** (20 mg) and **5** (35 mg). Fr.5 (8.0 g) was subjected to silica gel chromatography (100 g, 200–300 mesh) eluted with a CHCl_3_/Me_2_CO (100:0, 90:10, 80:20, *v/v*, each 2 L) gradient to afford six fractions (Frs.5a–f). Sub-fractions (5a–f) were respectively chromatographed through MCI (MeOH/Water: 10:90–70:30), Sephadex LH-20 (MeOH), and then further separated by silica gel chromatography with petroleum ether/Me_2_CO (70:30) and CHCl_3_/Me_2_CO (80:20) to furnish compounds **1** (15 mg), **6** (5 mg) and **7** (15 mg).

*p-Mentha-1(7), 8-dien-2-O-β-d-glucoside* (**1**). White amorphous powder; 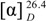 +4.7 (*c* 0.102, MeOH); IR (KBr) *v*_max_ 3423, 2923, 1643, 1437, 1074, 1020 cm^−1^; ^1^H- and ^13^C-NMR spectral data, see Table 1; HRESIMS: *m/z* 337.1622 (calcd. for C_16_H_26_O_6_Na, 337.1627).

*trans-2,4-Dihydroxy-2,4-dimethyl-trans-1-acetic acid γ-lactone* (**2**). White amorphous powder; 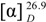 +133.3 (*c* 0.105, MeOH); IR (KBr) *v*_max_ 3446, 2972, 2940, 1746, 1378, 1268, 1105 cm^−1^; ^1^H- and ^13^C-NMR spectral data, see Table 2; HRESIMS: *m/z* 207.0999 (calcd. for C_1__0_H_16_O_3_Na, 207.0997).

*trans-2,4-Dihydroxy-2,4-dimethyl-cis-1-acetic acid γ-lactone* (**3**). White amorphous powder; 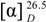 +54.5 (*c* 0.101, MeOH); IR (KBr) *v*_max_ 3473, 2924, 2852, 1735, 1382, 1269, 1100 cm^−1^; ^1^H- and ^13^C-NMR spectral data, see Table 2; HRESIMS: *m/z* 207.0994 (calcd. for C_1__0_H_16_O_3_Na, 207.0997).

## 4. Conclusions

This work was part of a series of investigations on anti-microbial compounds obtained from plants of the genus *Illicium*. Compounds **1** and **2** were found to be new monoterpenes, and the other five compounds were found for the first time in *I. lanceolatum*. The compounds **1**–**7** showed no inhibitory effects on the growth of the tested microbial organisms at less than 1.0 mg/mL *in vitro*.

## Supplementary Materials

Supplementary materials can be accessed at: http://www.mdpi.com/1420-3049/18/10/11866/s1.
