# Supplementary material for: Two New Monoterpenes from the Fruits of Illicium lanceolatum
Source: Molecules. 2013 Sep 26;18(10):11866–72. doi: 10.3390/molecules181011866 (PMC6269894; doi:10.3390/molecules181011866)
Supplement: Supplementary file 1 [file molecules-18-11866-s001.pdf]

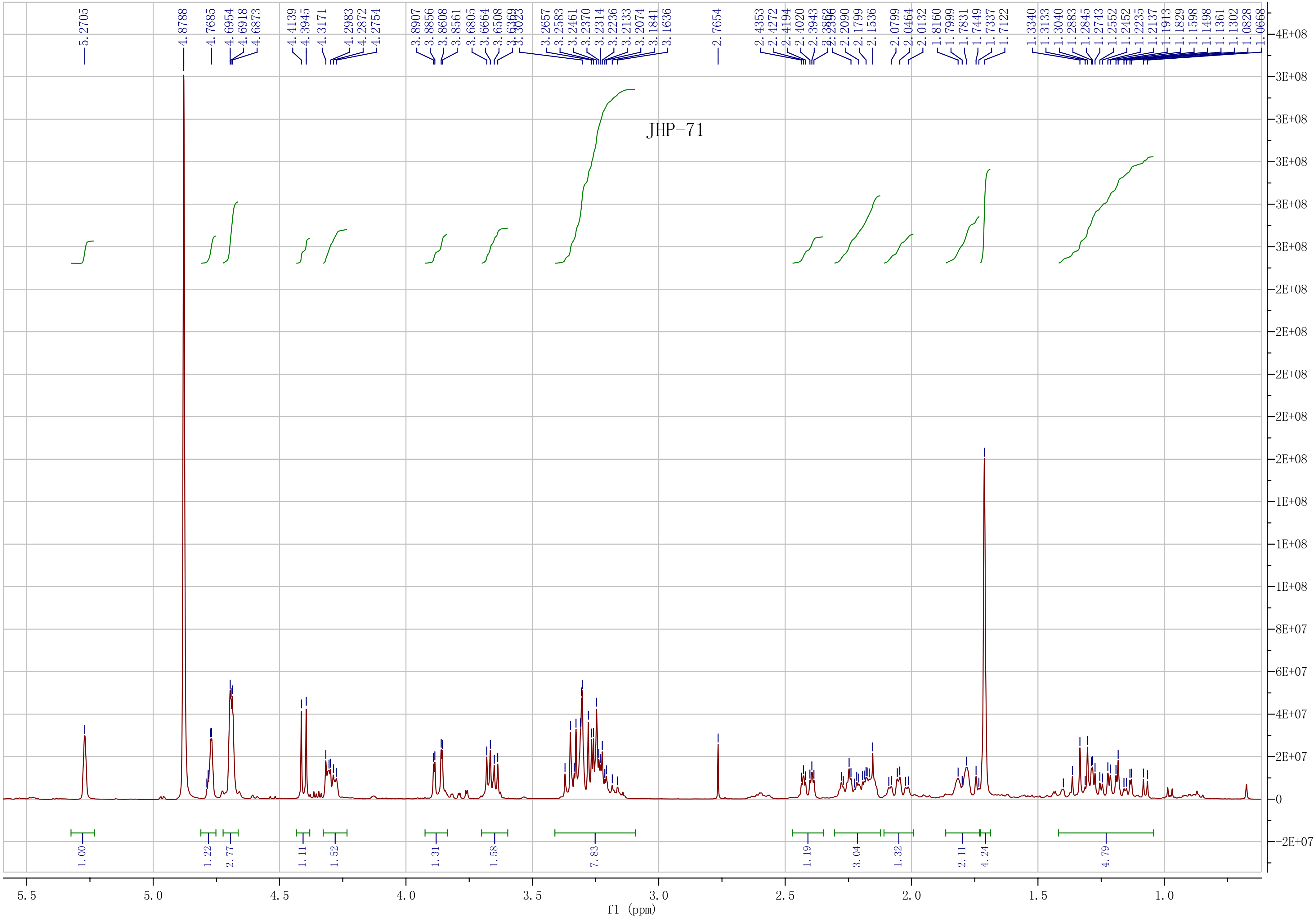

JHP-71
f1 (ppm)

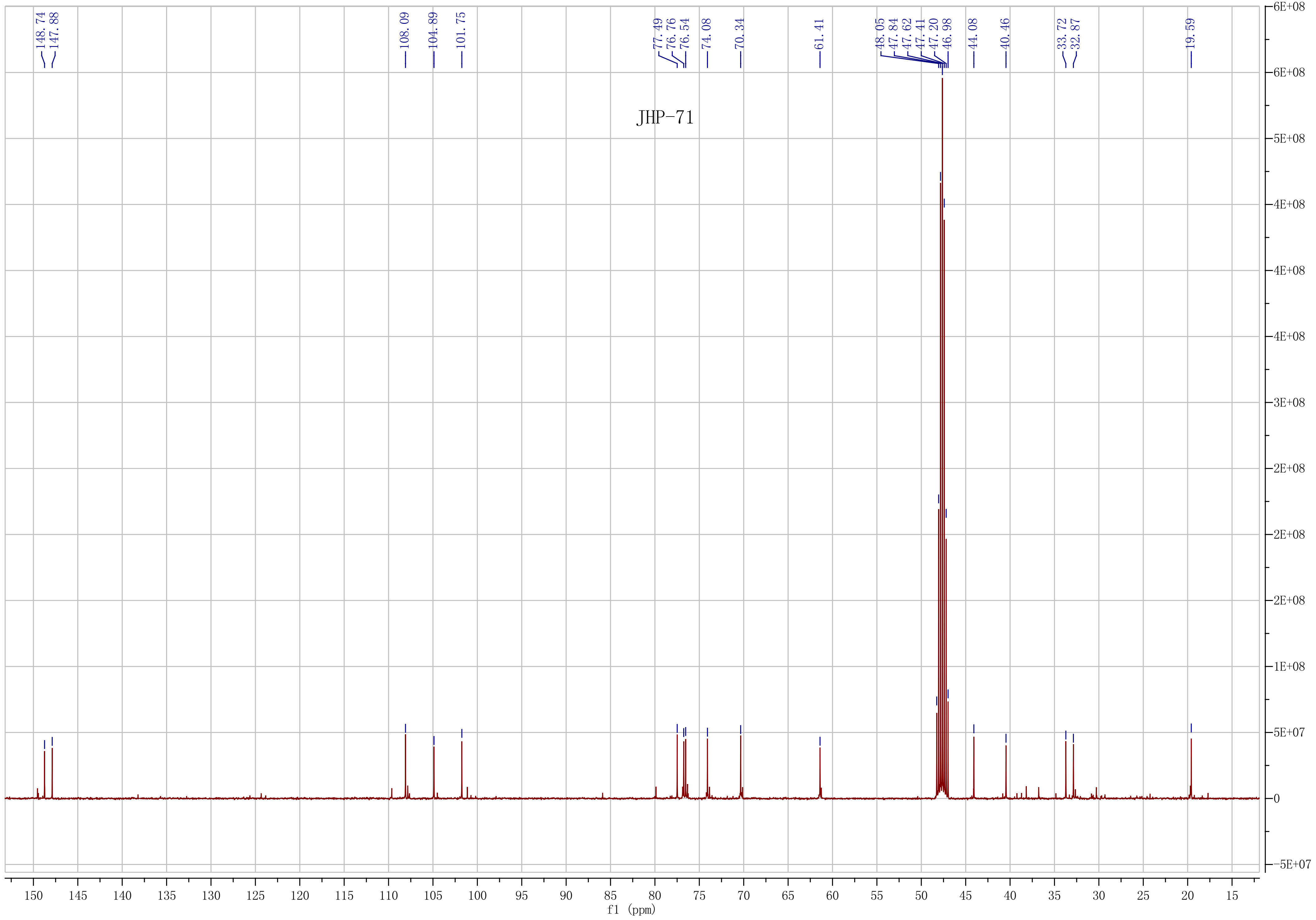

148.74
147.88
108.09
104.89
101.75
77.49
76.76
76.54
74.08
70.34
61.41
48.05
47.84
47.62
47.41
47.20
46.98
44.08
40.46
33.72
32.87
19.59
JHP-71
6E+08
6E+08
5E+08
4E+08
4E+08
4E+08
3E+08
2E+08
2E+08
2E+08
1E+08
5E+07
0
−5E+07
150
145
140
135
130
125
120
115
110
105
100
95
90
85
80
75
70
65
60
55
50
45
40
35
30
25
20
15
f1 (ppm)

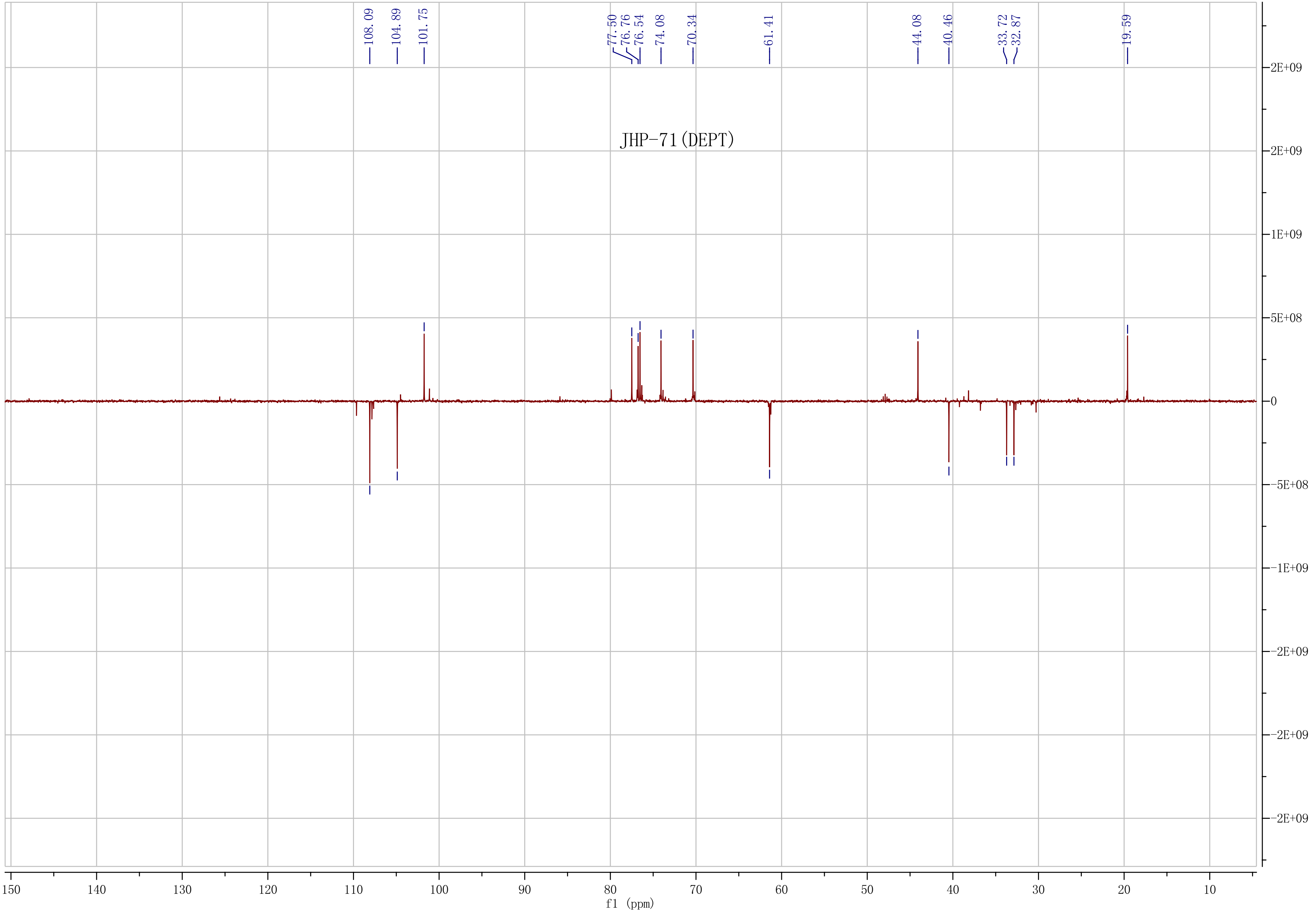

JHP-71(DEPT)
108.09
104.89
101.75
77.50
76.76
76.54
74.08
70.34
61.41
44.08
40.46
33.72
32.87
19.59
2E+09
2E+09
1E+09
5E+08
0
-5E+08
-1E+09
-2E+09
-2E+09
-2E+09
150
140
130
120
110
100
90
80
70
60
50
40
30
20
10
f1 (ppm)

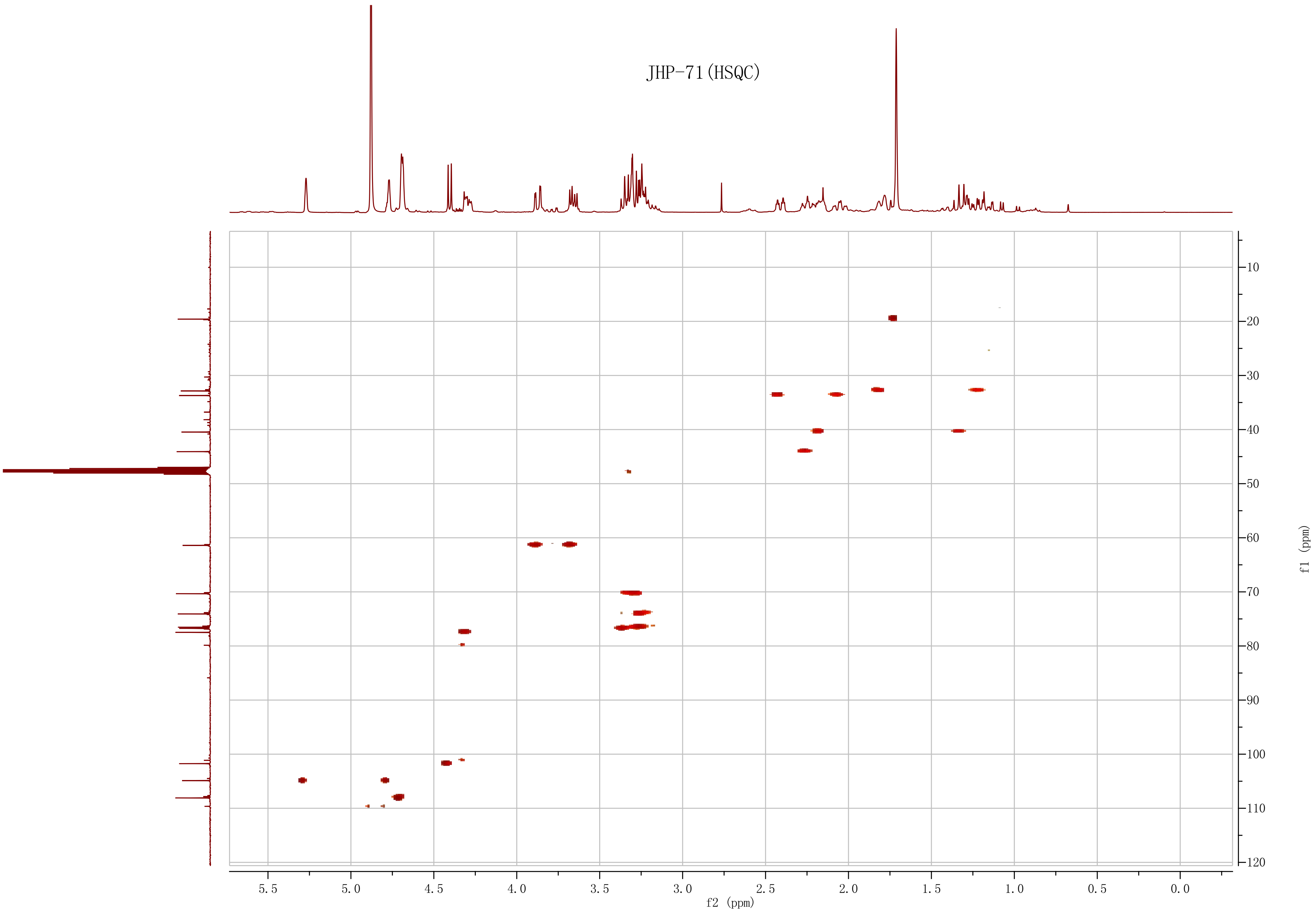

JHP-71(HSQC)
f2 (ppm)
f1 (ppm)
5.5
5.0
4.5
4.0
3.5
3.0
2.5
2.0
1.5
1.0
0.5
0.0
10
20
30
40
50
60
70
80
90
100
110
120

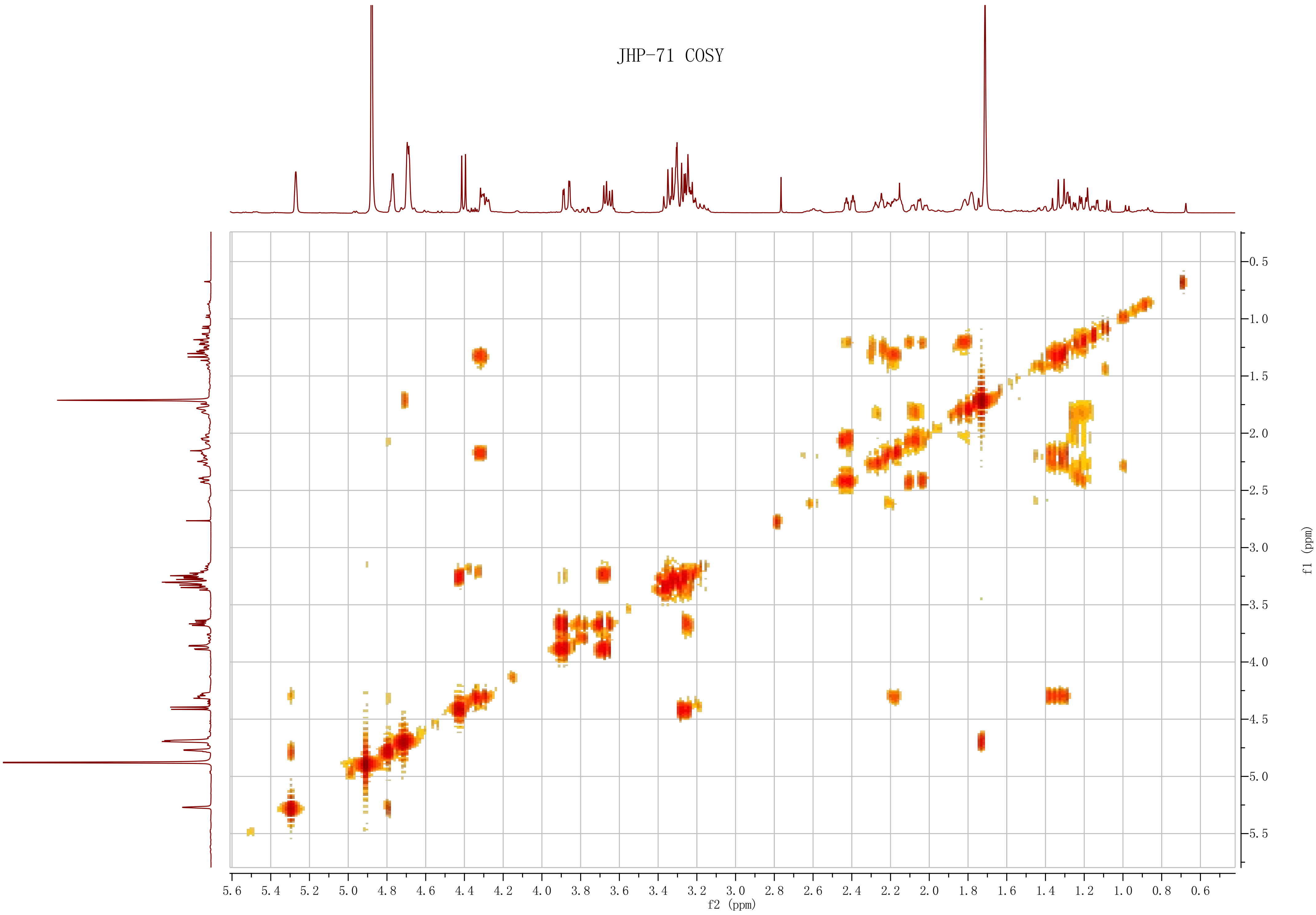
JHP-71 COSY
f2 (ppm)
f1 (ppm)
5.6 5.4 5.2 5.0 4.8 4.6 4.4 4.2 4.0 3.8 3.6 3.4 3.2 3.0 2.8 2.6 2.4 2.2 2.0 1.8 1.6 1.4 1.2 1.0 0.8 0.6
0.5 1.0 1.5 2.0 2.5 3.0 3.5 4.0 4.5 5.0 5.5

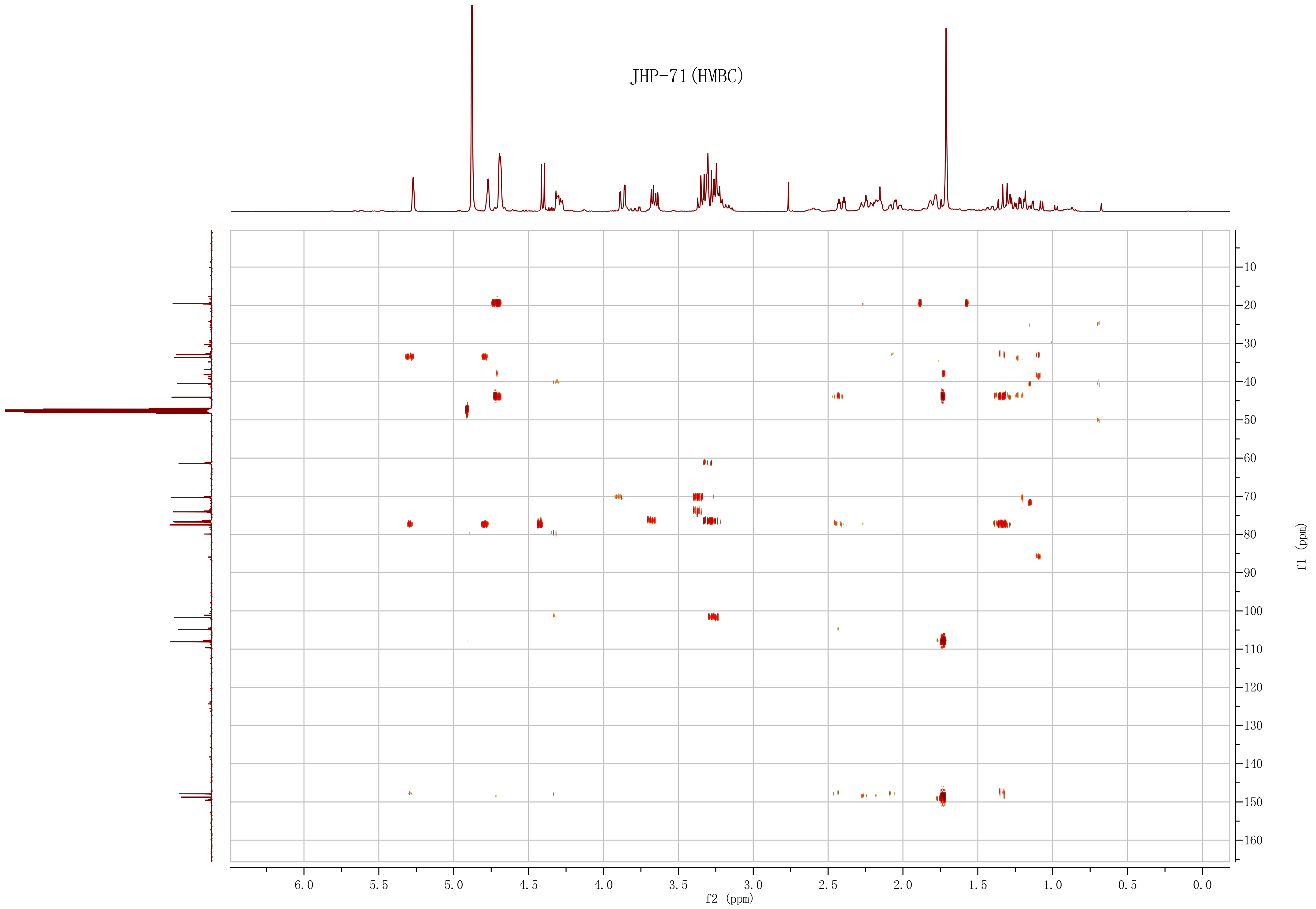
JHP-71(HMBC)
f2 (ppm)
f1 (ppm)
6.0
5.5
5.0
4.5
4.0
3.5
3.0
2.5
2.0
1.5
1.0
0.5
0.0
10
20
30
40
50
60
70
80
90
100
110
120
130
140
150
160

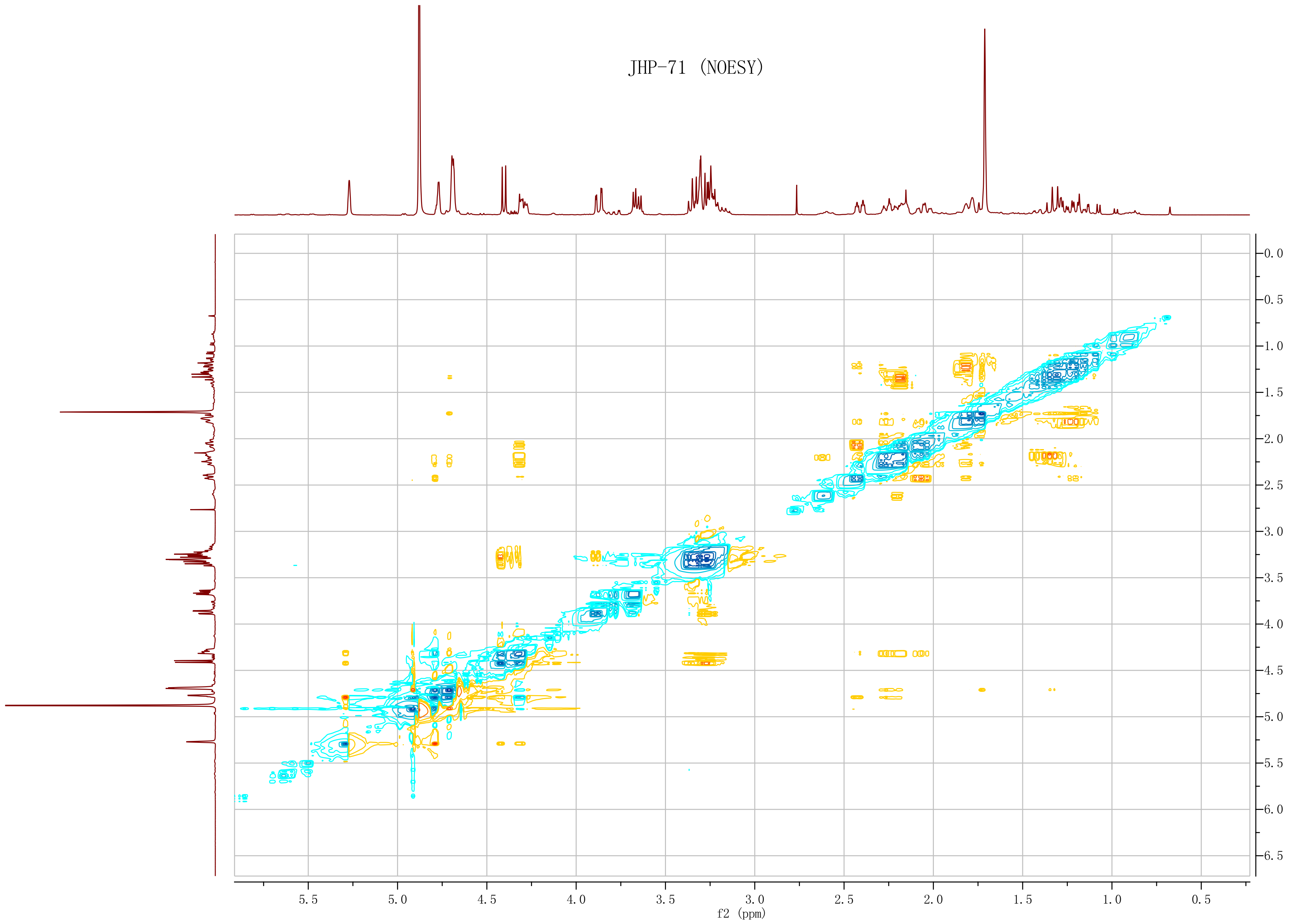
JHP-71 (NOESY)
f2 (ppm)
f1 (ppm)
5.5
5.0
4.5
4.0
3.5
3.0
2.5
2.0
1.5
1.0
0.5
0.0
6.0
6.5

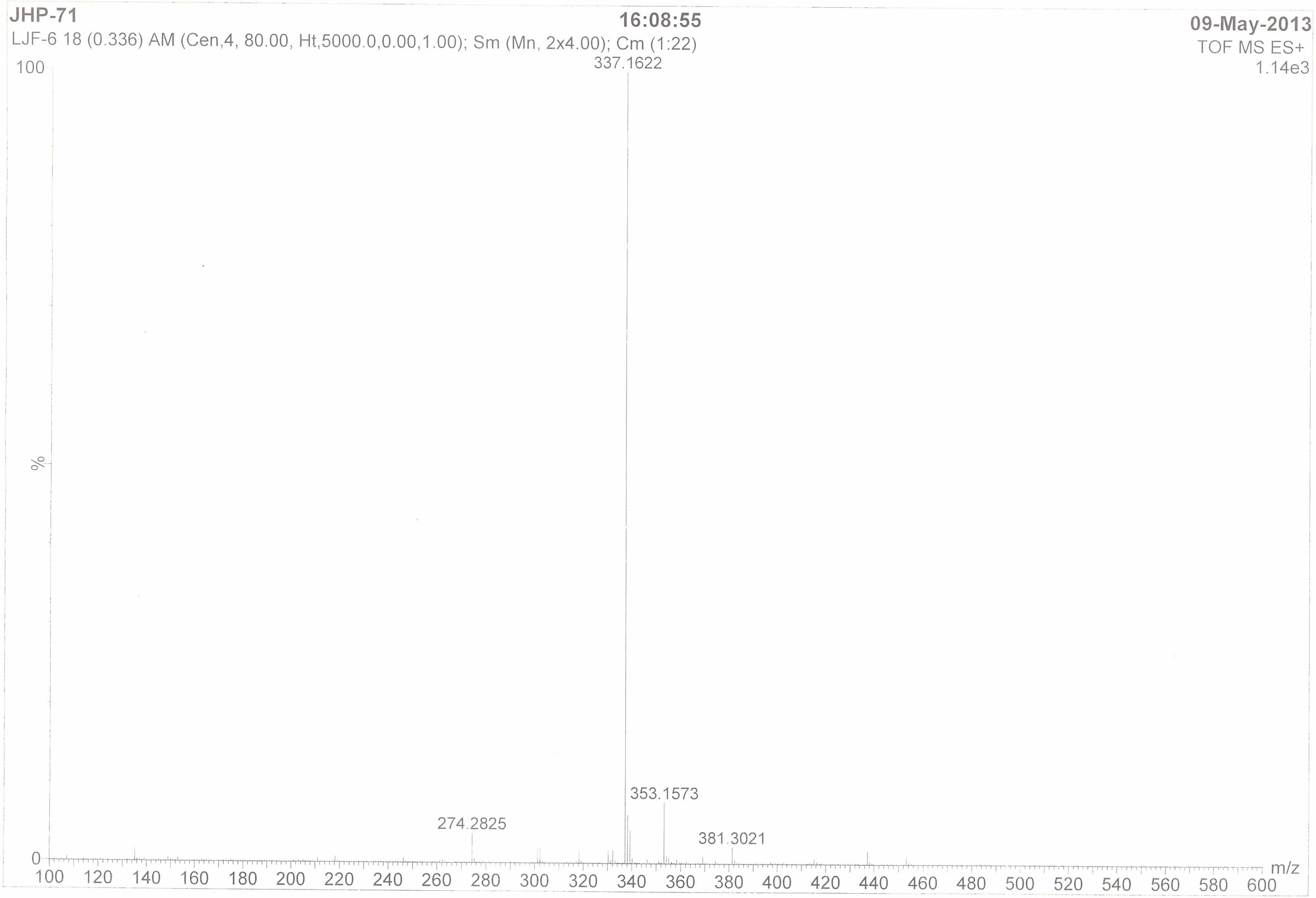
JHP-71
16:08:55
09-May-2013
LJF-6 18 (0.336) AM (Cen,4, 80.00, Ht,5000.0,0.00,1.00); Sm (Mn, 2x4.00); Cm (1:22)
TOF MS ES+
1.14e3
100
%
0
337.1622
353.1573
274.2825
381.3021
100 120 140 160 180 200 220 240 260 280 300 320 340 360 380 400 420 440 460 480 500 520 540 560 580 600
m/z

Elemental Composition Report

Single Mass Analysis
Tolerance = 5.0 PPM / DBE: min = -1.5, max = 50.0
Selected filters: None

Monoisotopic Mass, Even Electron Ions
59 formula(e) evaluated with 1 results within limits (all results (up to 1000) for each mass)
Elements Used:
C: 0-30 H: 0-1000 O: 0-6 Na: 0-1

JHP-71 16:08:55 09-May-2013
LJF-6 18 (0.336) AM (Cen,4, 80.00, Ht,5000.0,0.00,1.00); Sm (Mn, 2x4.00); Cm (1:22) TOF MS ES+ 1.14e3

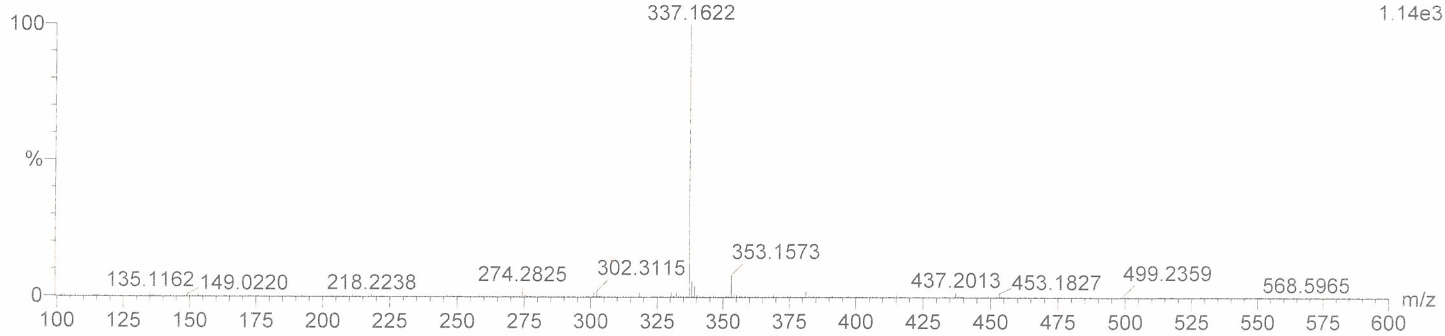

Minimum:				-1.5		
Maximum:		5.0	5.0	50.0		
Mass	Calc. Mass	mDa	PPM	DBE	i-FIT	Formula
337.1622	337.1627	-0.5	-1.5	3.5	95.0	C16 H26 O6 Na

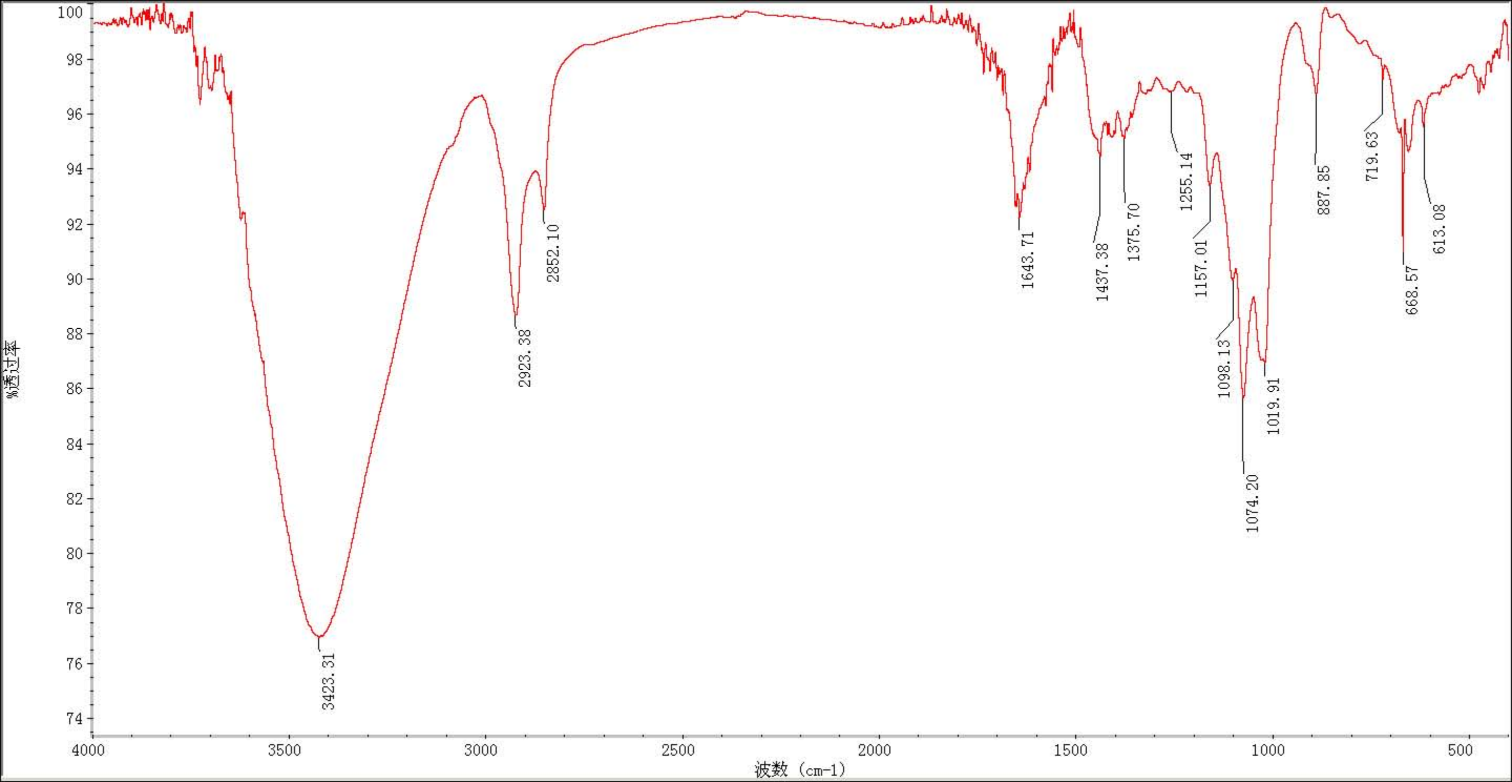

%透过率
波数 (cm-1)
3423.31
2923.38
2852.10
1643.71
1437.38
1375.70
1255.14
1157.01
1098.13
1074.20
1019.91
887.85
719.63
668.57
613.08

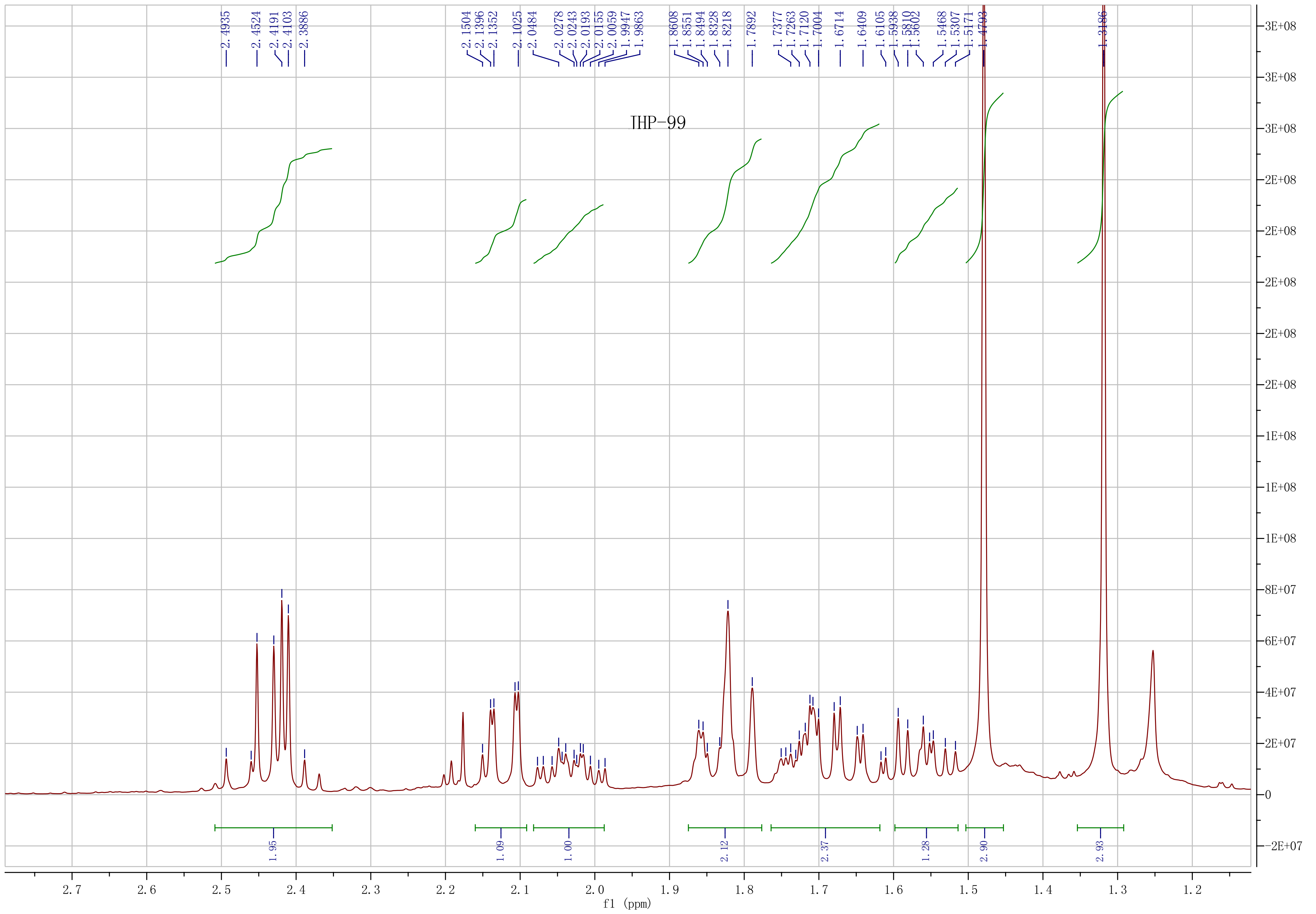

IHP-99
f1 (ppm)

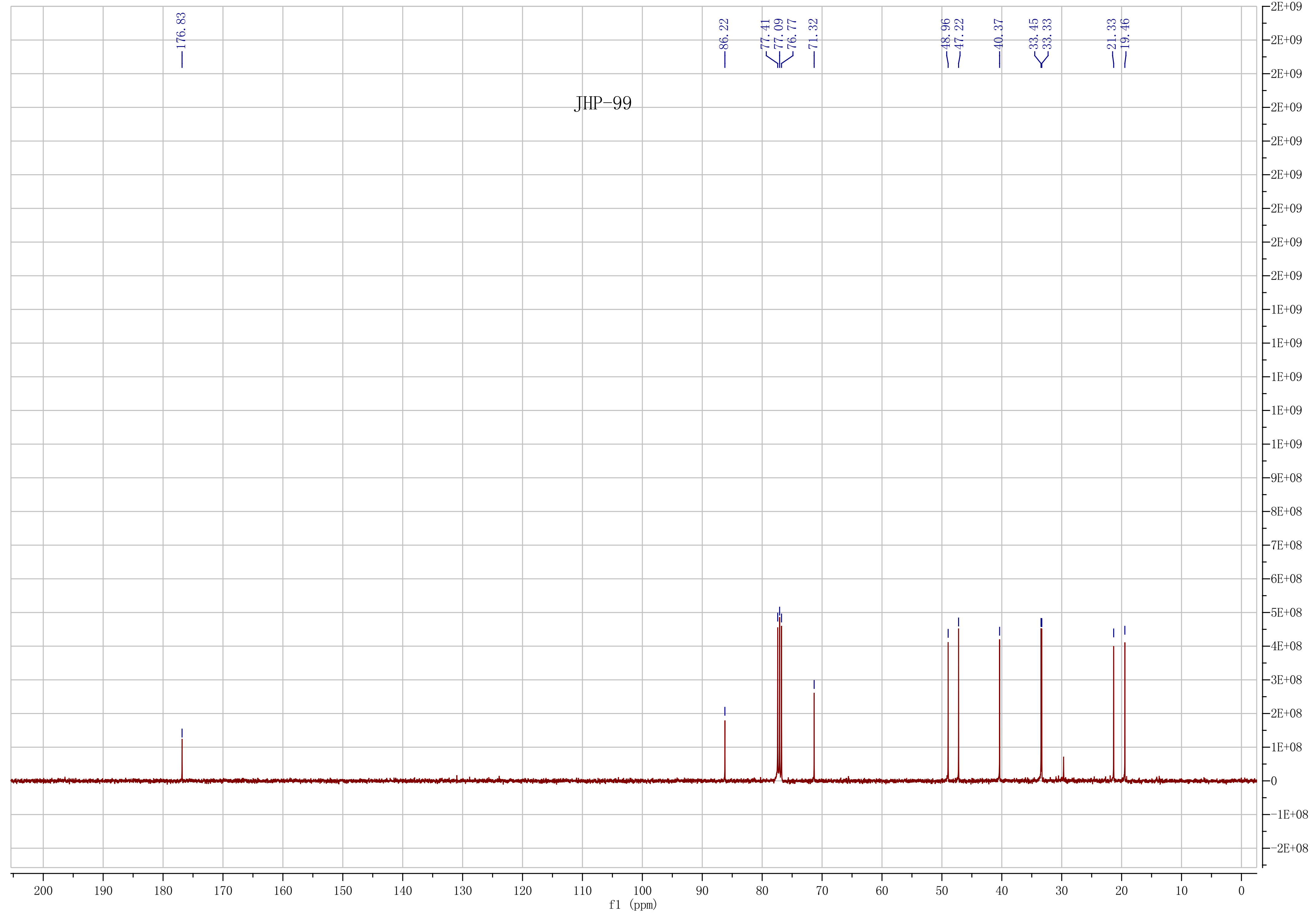
JHP-99
176.83
86.22
77.41
77.09
76.77
71.32
48.96
47.22
40.37
33.45
33.33
21.33
19.46
f1 (ppm)

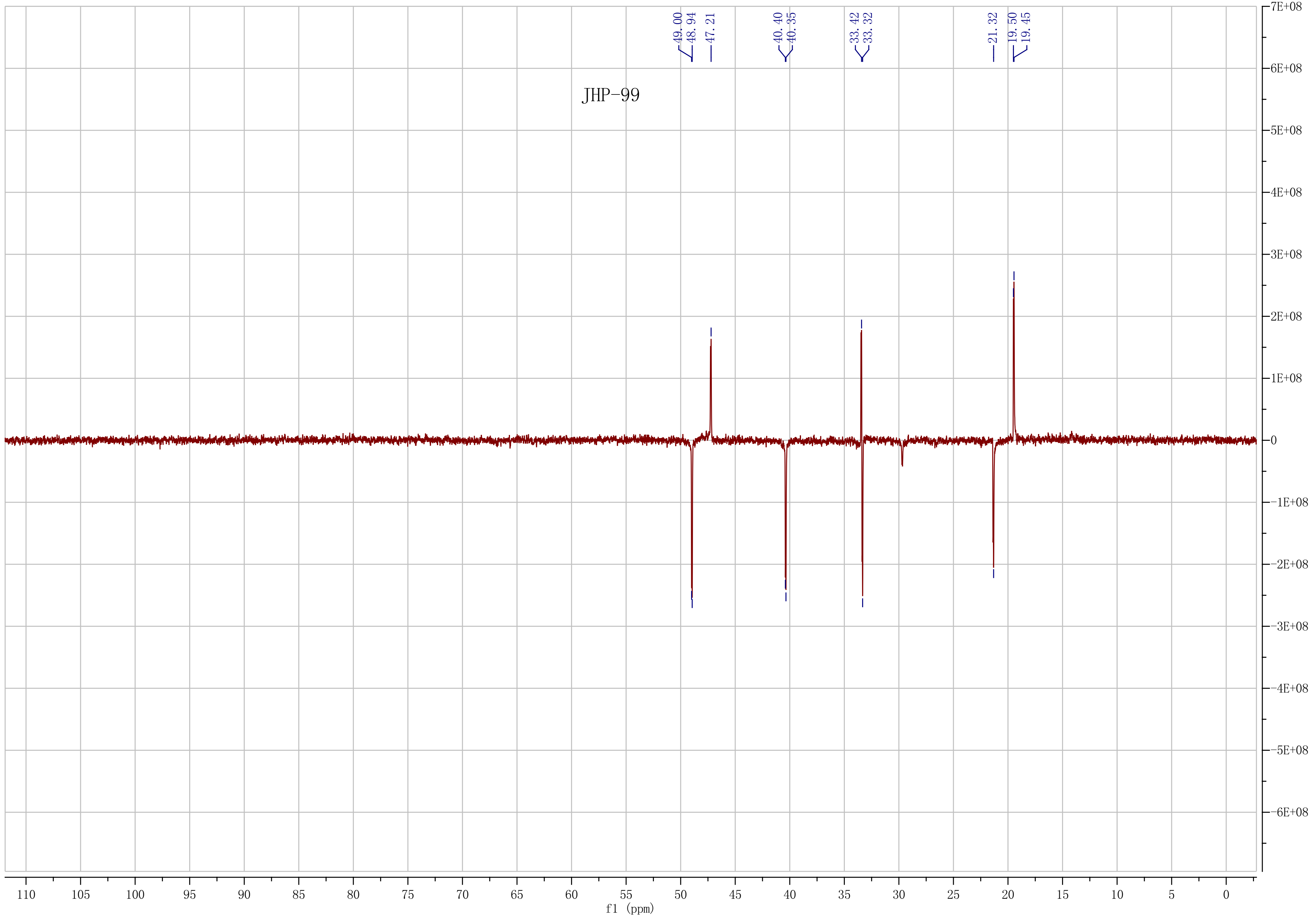
49.00
48.94
47.21
40.40
40.35
33.42
33.32
21.32
19.50
19.45
JHP-99
f1 (ppm)

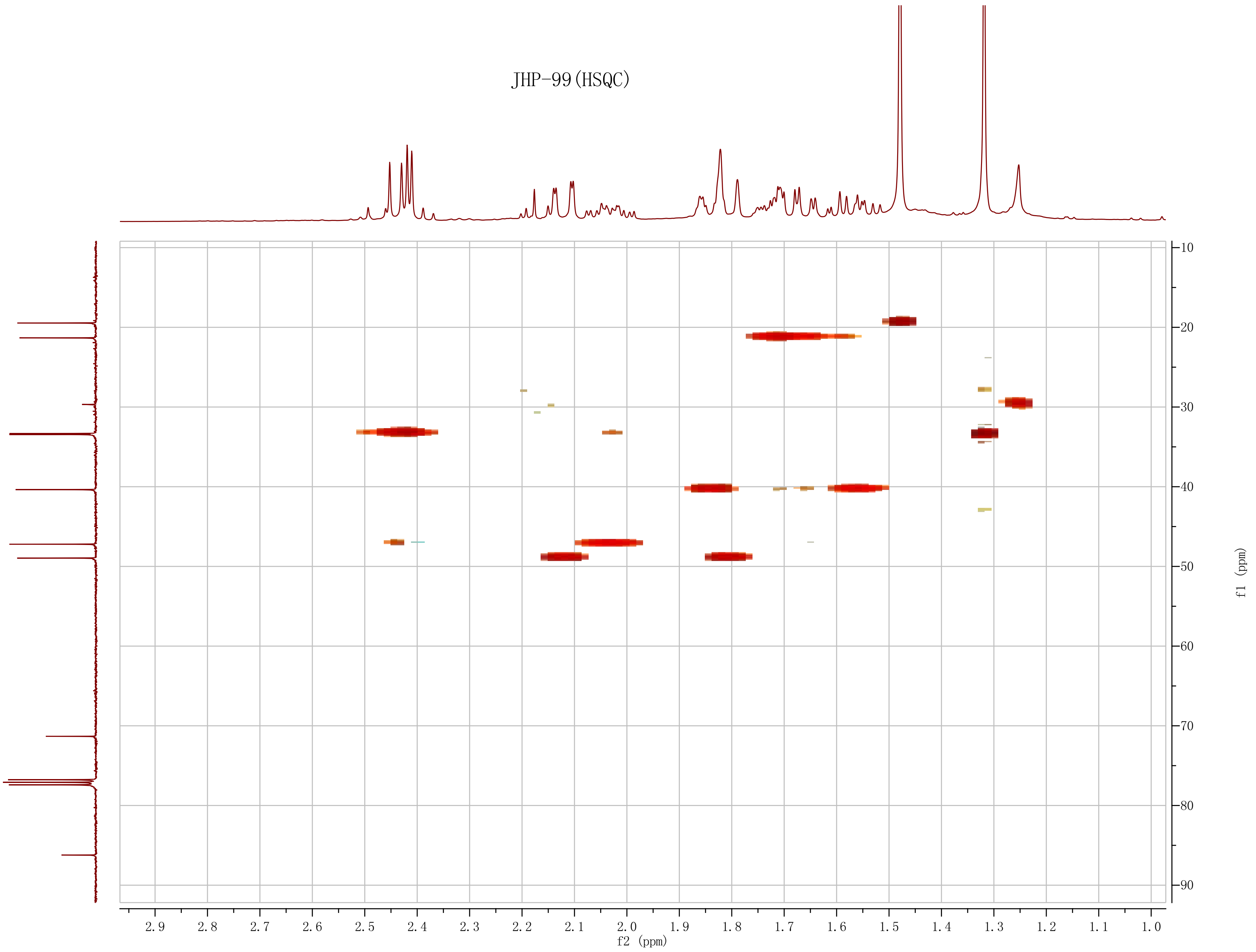

JHP-99(HSQC)
2.9
2.8
2.7
2.6
2.5
2.4
2.3
2.2
2.1
2.0
1.9
1.8
1.7
1.6
1.5
1.4
1.3
1.2
1.1
1.0
f2 (ppm)
10
20
30
40
50
60
70
80
90
f1 (ppm)

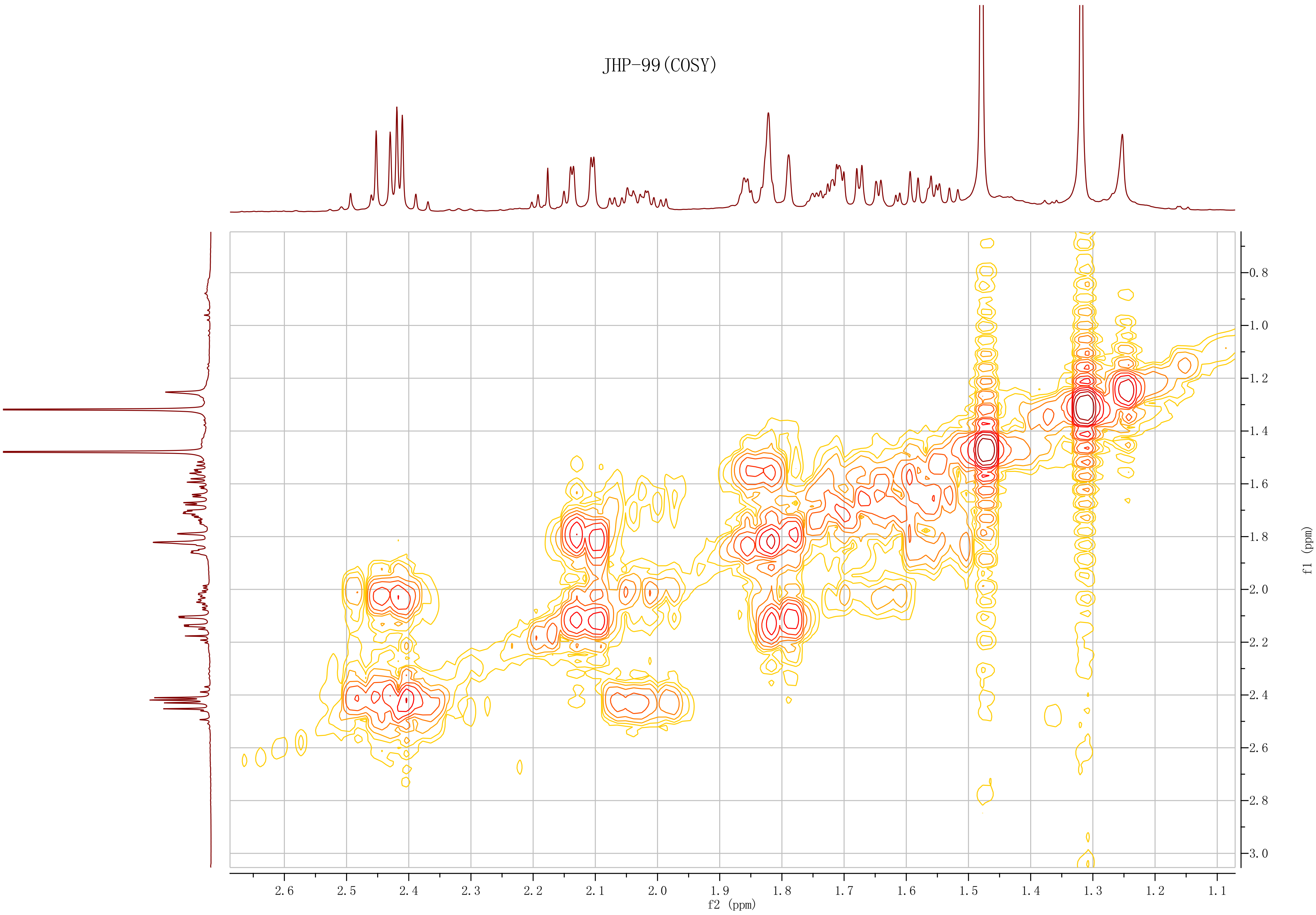
JHP-99(COSY)
f2 (ppm)
f1 (ppm)
2.6
2.5
2.4
2.3
2.2
2.1
2.0
1.9
1.8
1.7
1.6
1.5
1.4
1.3
1.2
1.1
0.8
1.0
1.2
1.4
1.6
1.8
2.0
2.2
2.4
2.6
2.8
3.0

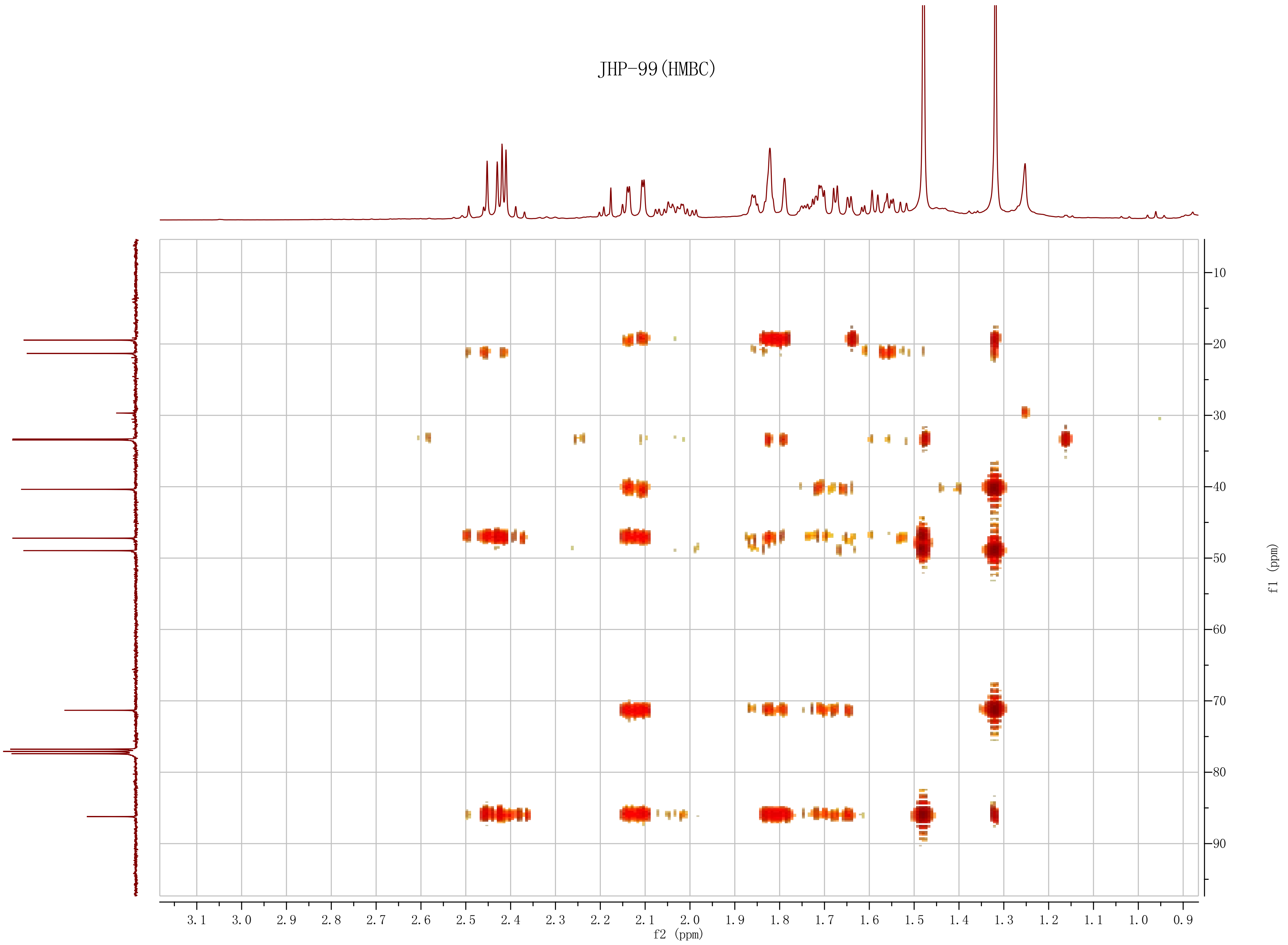

JHP-99(HMBC)
3.1
3.0
2.9
2.8
2.7
2.6
2.5
2.4
2.3
2.2
2.1
2.0
1.9
1.8
1.7
1.6
1.5
1.4
1.3
1.2
1.1
1.0
0.9
f2 (ppm)
10
20
30
40
50
60
70
80
90
f1 (ppm)

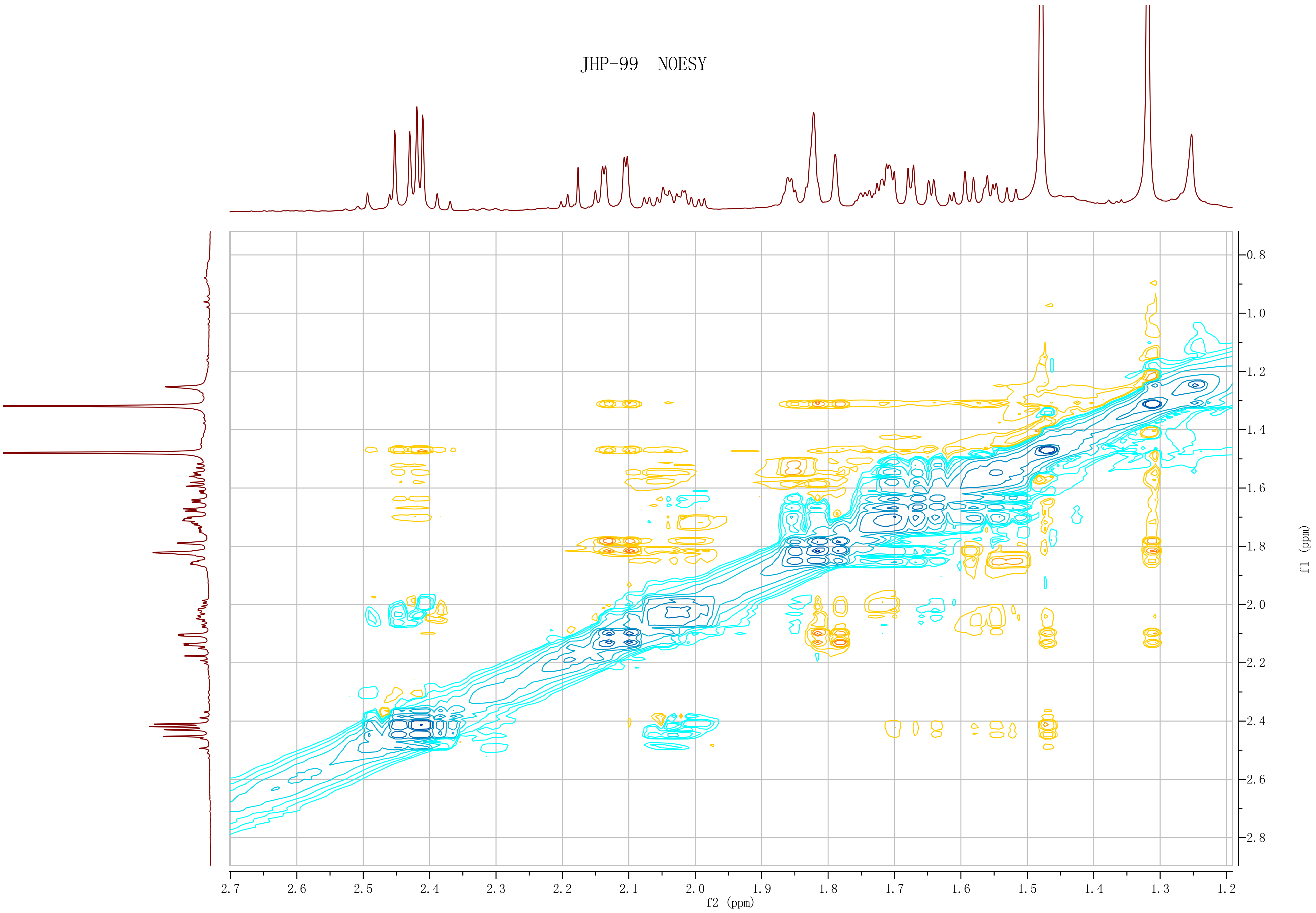
JHP-99 NOESY
f2 (ppm)
f1 (ppm)
2.7
2.6
2.5
2.4
2.3
2.2
2.1
2.0
1.9
1.8
1.7
1.6
1.5
1.4
1.3
1.2
0.8
1.0
1.2
1.4
1.6
1.8
2.0
2.2
2.4
2.6
2.8

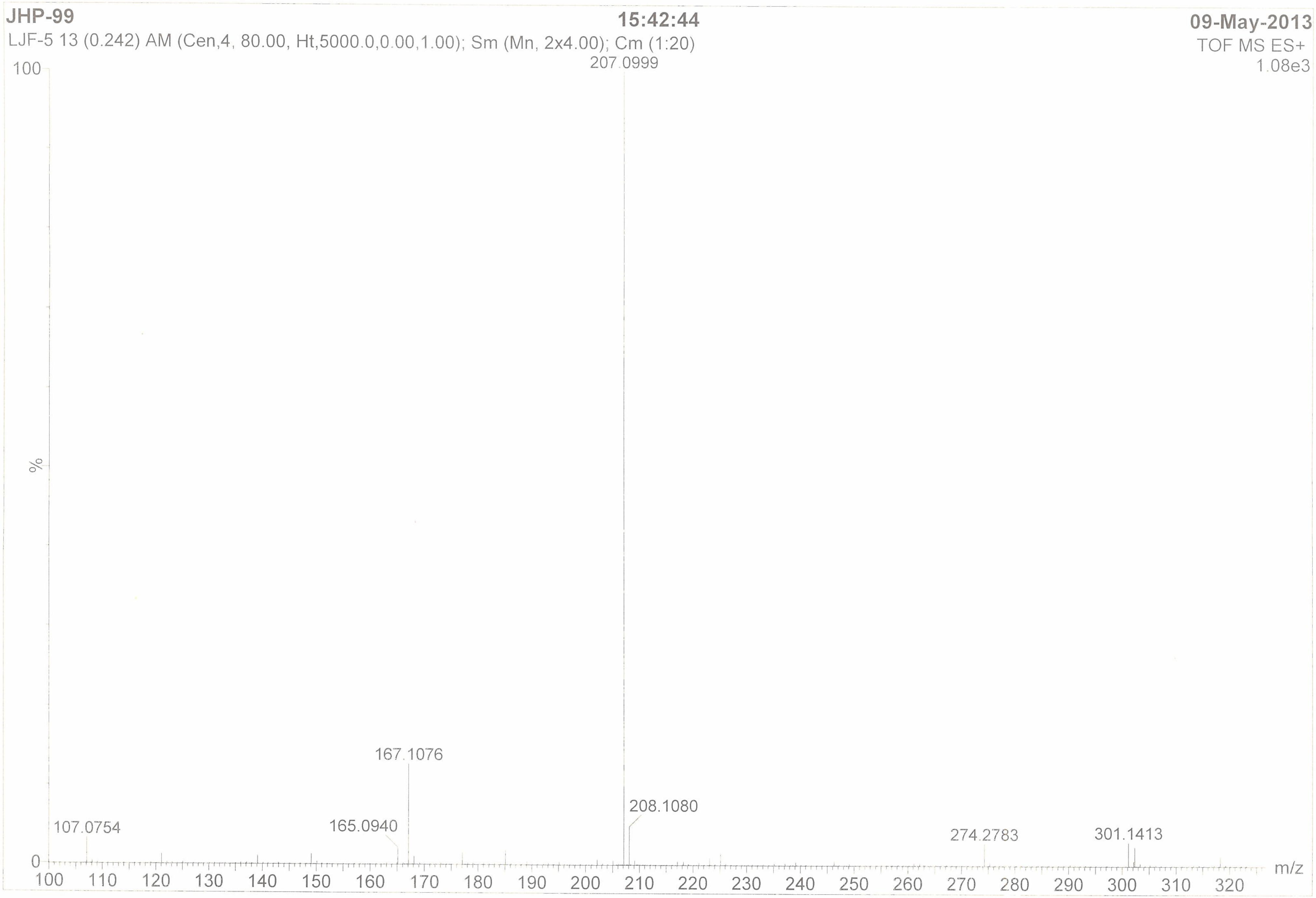

JHP-99
15:42:44
09-May-2013
LJF-5 13 (0.242) AM (Cen,4, 80.00, Ht,5000.0,0.00,1.00); Sm (Mn, 2x4.00); Cm (1:20)
TOF MS ES+
1.08e3
100
%
0
207.0999
167.1076
208.1080
107.0754
165.0940
274.2783
301.1413
100 110 120 130 140 150 160 170 180 190 200 210 220 230 240 250 260 270 280 290 300 310 320
m/z

Single Mass Analysis

Tolerance = 5.0 PPM / DBE: min = -1.5, max = 50.0
Selected filters: None

Monoisotopic Mass, Even Electron Ions
31 formula(e) evaluated with 1 results within limits (all results (up to 1000) for each mass)
Elements Used:
C: 0-30 H: 0-1000 O: 0-4 Na: 0-1

JHP-99 15:42:44 09-May-2013
LJF-5 13 (0.242) AM (Cen,4, 80.00, Ht,5000.0,0.00,1.00); Sm (Mn, 2x4.00); Cm (1:20) TOF MS ES+ 1.08e3

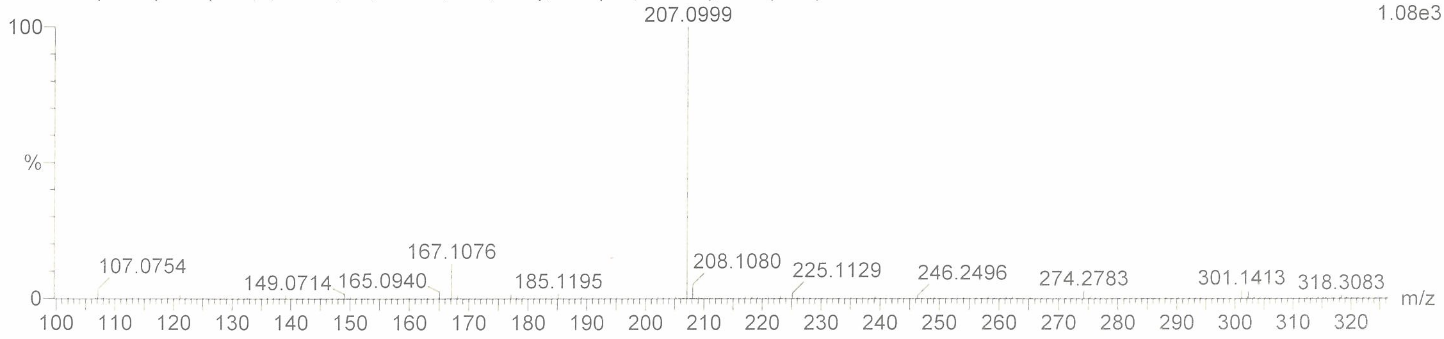

Minimum:				-1.5		
Maximum:		5.0	5.0	50.0		
Mass	Calc. Mass	mDa	PPM	DBE	i-FIT	Formula
207.0999	207.0997	0.2	1.0	2.5	41.1	C10 H16 O3 Na

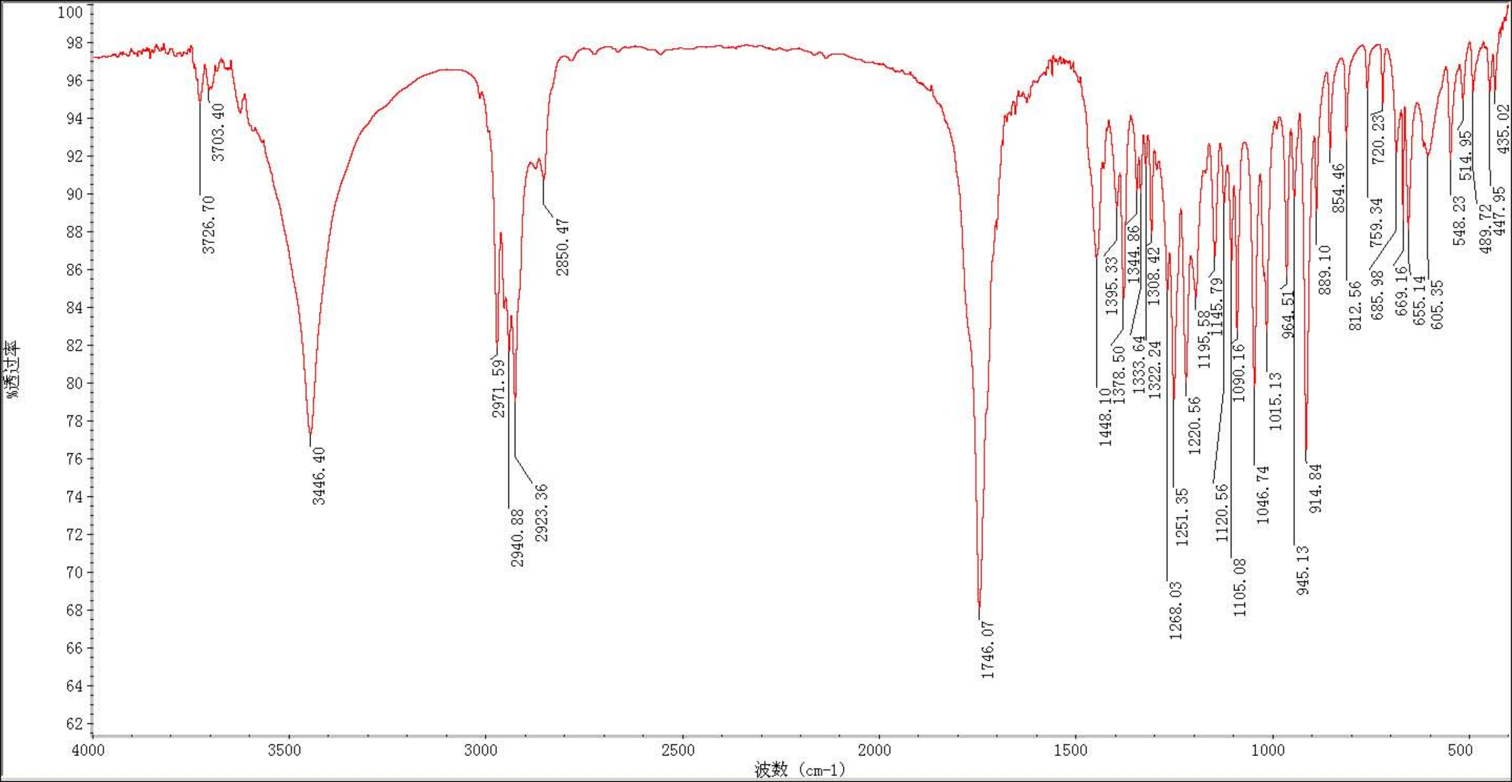

%透过率
波数 (cm-1)
3726.70
3703.40
3446.40
2971.59
2940.88
2923.36
2850.47
1746.07
1448.10
1395.33
1378.50
1344.86
1333.64
1322.24
1308.42
1268.03
1251.35
1220.56
1195.58
1145.79
1120.56
1105.08
1090.16
1046.74
1015.13
964.51
945.13
914.84
889.10
854.46
812.56
759.34
720.23
685.98
669.16
655.14
605.35
548.23
514.95
489.72
447.95
435.02

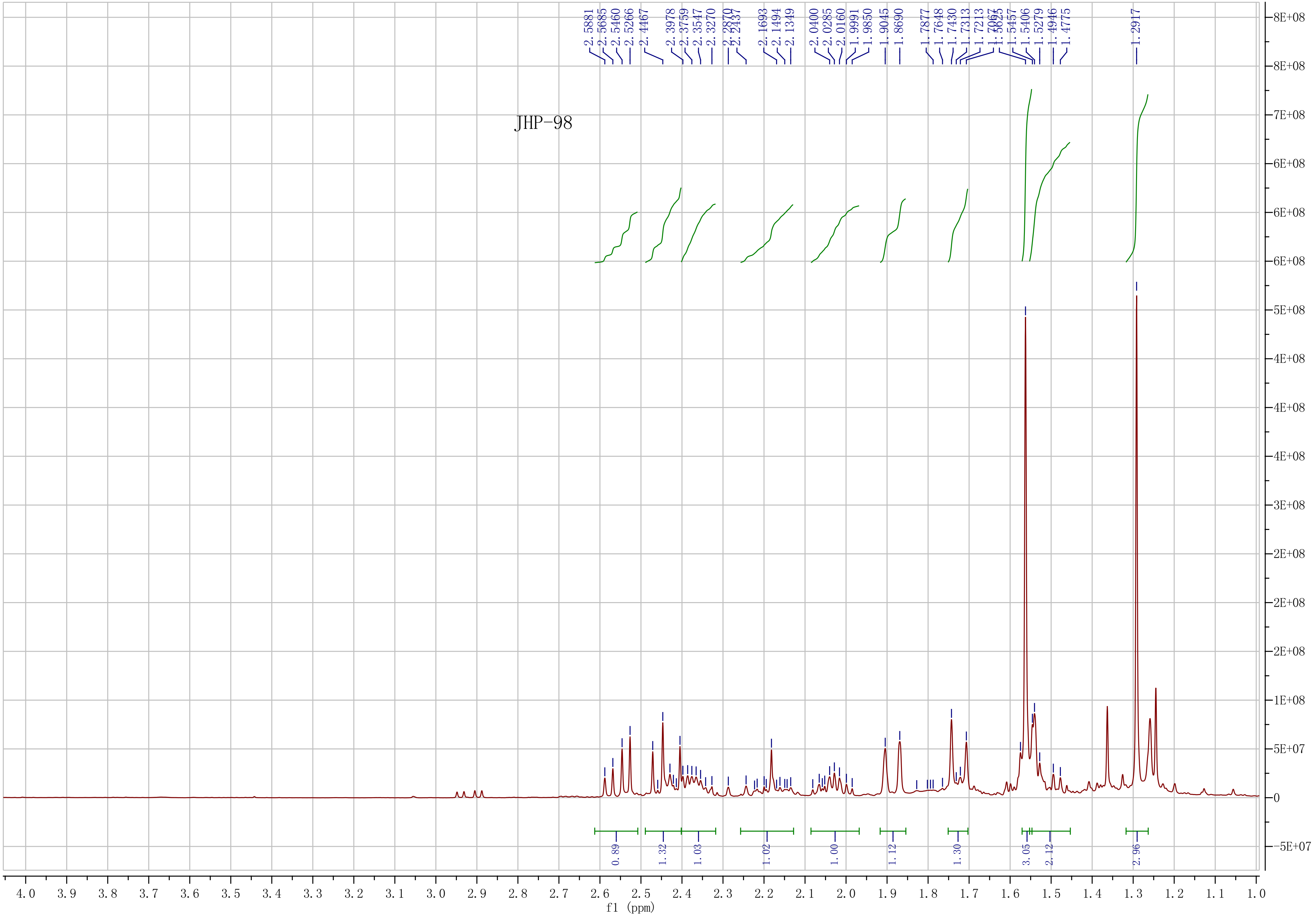
JHP-98
2.5881
2.5685
2.5460
2.5266
2.4467
2.3978
2.3759
2.3547
2.3270
2.2437
2.1693
2.1494
2.1349
2.0400
2.0285
2.0160
1.9991
1.9850
1.9045
1.8690
1.7877
1.7648
1.7430
1.7313
1.7213
1.5457
1.5406
1.5279
1.4946
1.4775
1.2917
0.89
1.32
1.03
1.02
1.00
1.12
1.30
3.05
2.12
2.96
f1 (ppm)

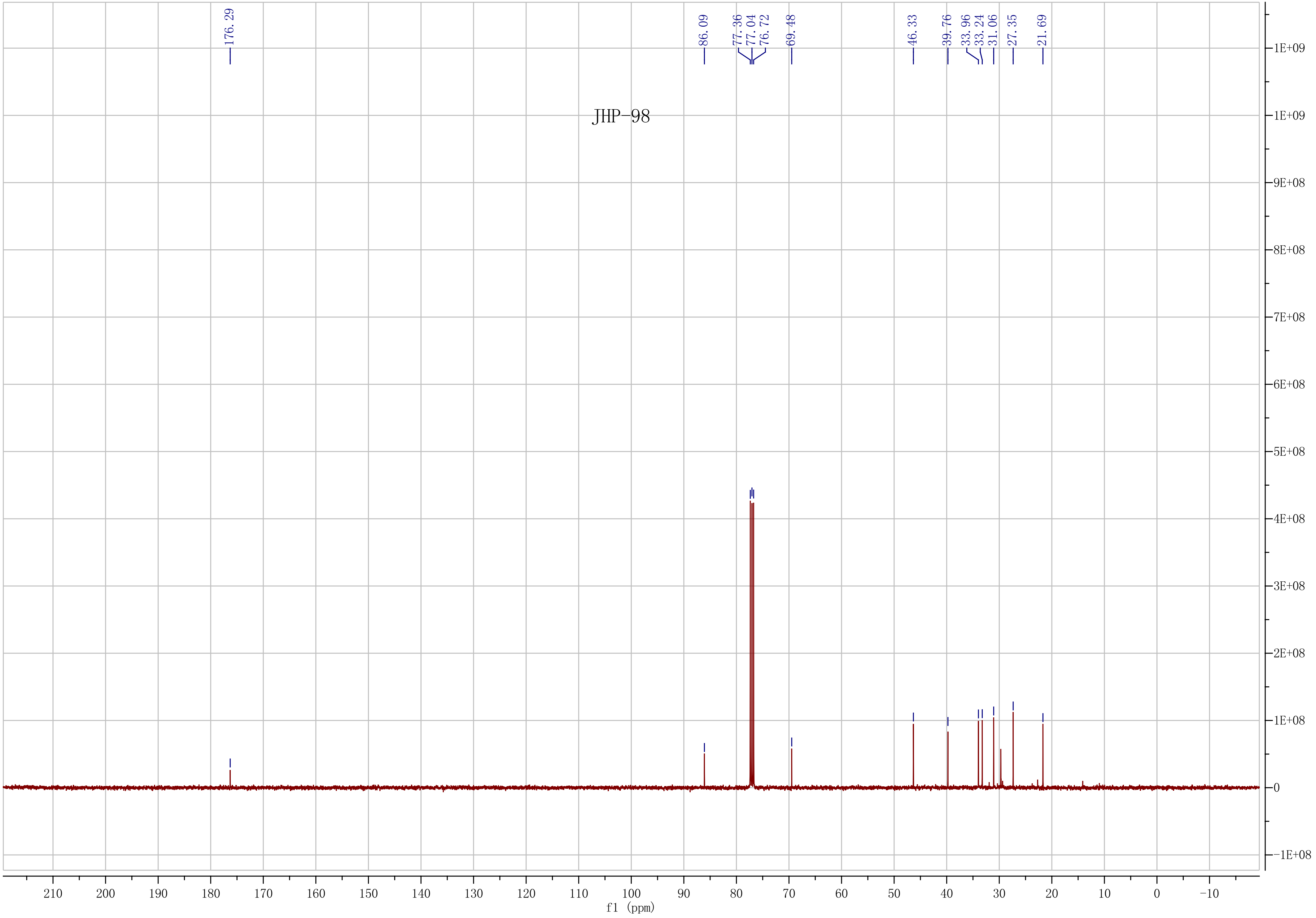

176.29
86.09
77.36
77.04
76.72
69.48
46.33
39.76
33.96
33.24
31.06
27.35
21.69
JHP-98
1E+09
1E+09
9E+08
8E+08
7E+08
6E+08
5E+08
4E+08
3E+08
2E+08
1E+08
0
-1E+08
210 200 190 180 170 160 150 140 130 120 110 100 90 80 70 60 50 40 30 20 10 0 -10
f1 (ppm)

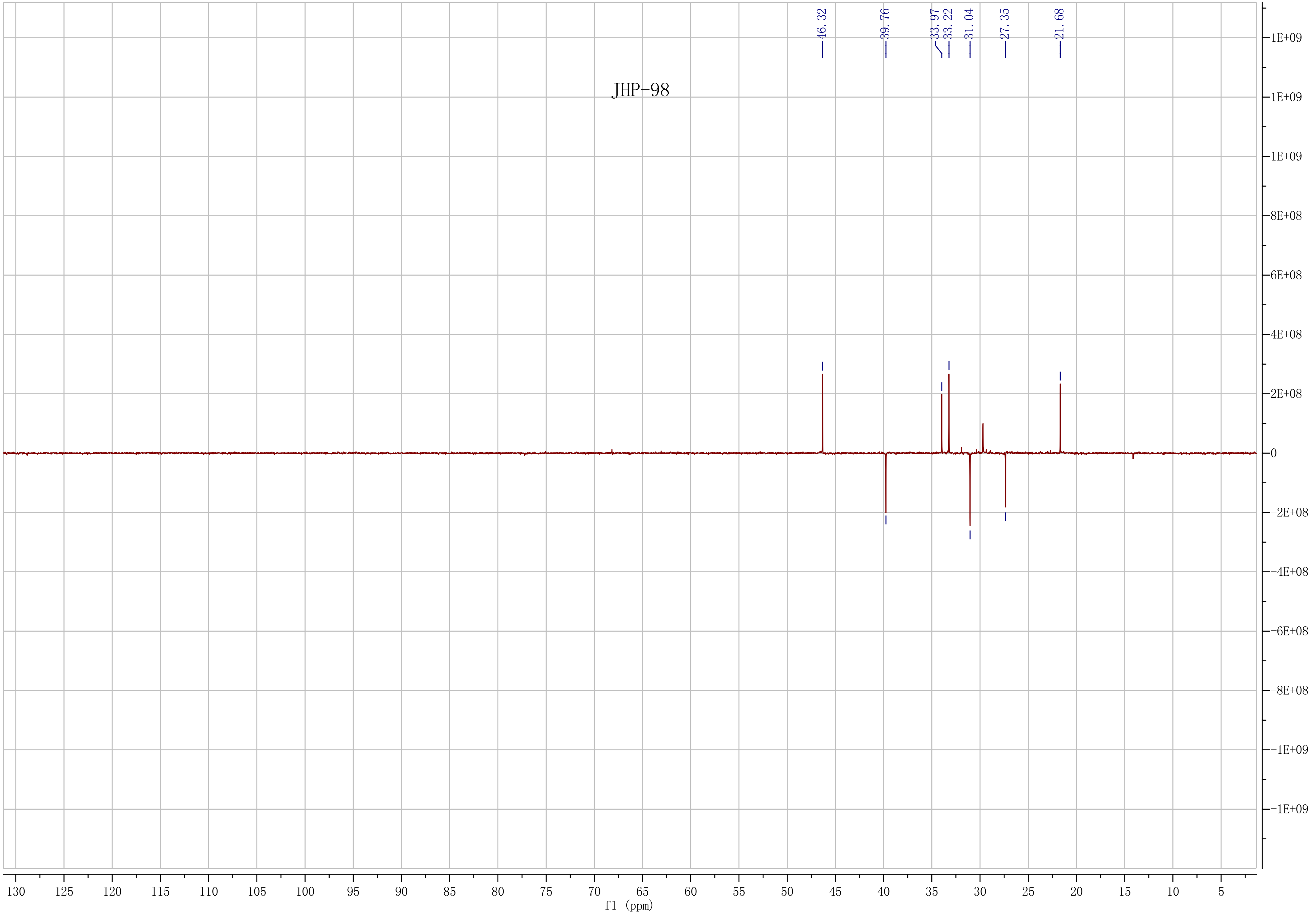
JHP-98
46.32
39.76
33.97
33.22
31.04
27.35
21.68
f1 (ppm)
